# Composition-Dependent Deformation and Shape-Memory Mechanisms of PETG/POE Blends: An All-Atom Molecular Dynamics Study

**DOI:** 10.3390/polym18141734

**Published:** 2026-07-15

**Authors:** Xiaoqing Feng, Jiangwen Chen, Lei Zhu, Chao Cao, Yunfei Cai, Xin Luo

**Affiliations:** 1 School of Advanced Manufacturing, Nanchang University, Nanchang 330031, China; 2 State Key Laboratory of Material Processing and Die and Mould Technology, School of Materials Science and Engineering, Huazhong University of Science and Technology, Wuhan 430074, China

**Keywords:** PETG/POE blends, shape-memory polymers, molecular dynamics, thermomechanical recovery, shape fixity and recovery

## Abstract

PETG/POE blends are thermo-responsive shape-memory systems with composition-dependent deformation and thermomechanical recovery behavior, but their molecular mechanism remains unclear. In this work, all-atom molecular dynamics simulations were performed for PETG/POE blends with different compositions. Model reliability was supported by density stabilization during equilibration and by reasonable agreement between simulated and experimental glass transition temperatures, with glass transition temperature deviations below 1.3%. Tensile simulations showed that increasing POE content reduced Young’s modulus from 1.81 to 1.10 GPa and yield stress from 0.251 to 0.144 GPa, indicating decreased stiffness and enhanced deformation accommodation. Free-volume and cavity analyses indicated tensile-induced packing loosening, cavity nucleation, and subsequent cavity growth and coalescence. Component-resolved interaction-energy decomposition and phase-resolved mean square displacement analyses showed strong PETG-related cohesive interactions, restricted PETG mobility at 200 K, enhanced POE mobility at 450 K, and relatively stronger PETG-POE interactions at intermediate compositions. These results help us to correlate blend composition, local structure, interaction-energy reorganization, chain conformation, and segmental mobility with fixation-recovery behavior. Under the present simulation protocol, PETG4/POE6 showed a relatively balanced response because of the compromise among rigidity, intermolecular interactions, cavity evolution, and thermally activated mobility.

## 1. Introduction

Thermo-responsive shape-memory polymers can be programmed into a temporary shape and recover their permanent shape upon heating, making them attractive for programmable polymer structures and 4D printing applications [1,2,3,4,5]. In amorphous thermoplastics, this response is primarily controlled by the glass transition temperature (*T*_g_), molecular packing, segmental mobility, and deformation-induced structural rearrangement [6,7,8]. Poly(ethylene terephthalate glycol) (PETG) is an amorphous copolyester with good transparency, dimensional stability, melt processability, and compatibility with fused deposition modeling [9,10,11,12]. Its relatively high *T*_g_ and rigid aromatic ester structure make PETG suitable for providing stiffness and temporary-shape fixation in thermo-responsive systems. However, the same rigid aromatic ester structure also limits ductility and chain mobility. As a result, neat PETG may be less favorable for large-strain programming and recovery [13,14].

The properties of PETG-based materials can be adjusted by incorporating flexible phases or functional components [15,16]. da Cunha et al. reported that PETG/TPU blends combine the stiffness and shape-fixity contribution of PETG with the recovery ability of the elastomeric TPU phase [10]. Lira et al. prepared supertough PETG/EGMA thermoplastic vulcanizates and achieved improved ductility while preserving shape-memory functionality [17]. Jie et al. investigated PETG-rich olefin blends and showed that blend composition strongly influenced phase morphology, mechanical response, and shape-memory behavior [18]. Ali et al. designed PETG-PBAT blends for 3D/4D printing and showed that the introduction of a flexible biodegradable polyester phase affected the thermal, mechanical, and shape-memory response of the blends [19]. Bouguermouh et al. studied PLA-PETG blends and found that their thermal, mechanical, and shape-memory properties were strongly composition-dependent [11]. Ali et al. also evaluated PETG-EVA systems and showed that the introduction of a flexible secondary phase affected printability, morphology, and shape-memory performance [12]. Liu and Chen further reported that HDPE-PETG blends exhibited composition-dependent mechanical relaxation and shape-memory behavior [14].

In shape-memory polymer blends, a rigid phase is generally responsible for temporary-shape fixation, whereas a flexible or elastomeric phase can enhance recovery and conformational reversibility [20,21,22,23,24,25]. Poly(ethylene-co-octene) (POE) is a thermoplastic polyolefin elastomer with a flexible saturated backbone, a low glass transition temperature, good elasticity, and excellent melt processability. In PETG/POE blends, PETG is expected to serve as the rigid phase and provide stiffness and temporary-shape fixation. POE may improve deformation tolerance, elastic recovery, and conformational reversibility [26]. Overall, previous studies suggest that the behavior of PETG-based thermo-responsive blends is governed by the coupling between rigid-phase fixation and flexible-phase-assisted recovery. PETG/POE blends are therefore also expected to exhibit pronounced composition-dependent behavior [10,17,18,26,27,28,29,30,31,32,33].

Recently, Cao et al. prepared thermo-responsive PETG/POE blends for fused deposition modeling-based 4D printing [26]. Their experimental results showed that increasing POE content changed the blend morphology, improved elongation at break, reduced tensile strength, and affected shape fixity and recovery behavior. Among the investigated compositions, PETG4/POE6 exhibited relatively balanced shape-memory performance. Compared with previous PETG-based blend studies, this system provides a useful experimental basis for discussing how a rigid copolyester phase and a flexible polyolefin phase jointly affect mechanical performance and fixation-recovery behavior. However, the molecular mechanism underlying this composition-dependent behavior remains insufficiently understood. Experimental characterization can correlate morphology, thermal transitions, and macroscopic properties. However, it cannot directly determine how PETG and POE differ in local mobility, intermolecular interactions, cavity formation, and conformational evolution during deformation and recovery. Molecular-level analysis is therefore needed to further examine how POE content affects local packing, intermolecular interactions, deformation coordination, temporary-shape fixation, segmental mobility, and thermal recovery in PETG/POE blends at the atomistic scale.

Molecular dynamics simulation provides a useful route for analyzing polymer blends at the molecular scale because it can track chain conformation, intermolecular interactions, local packing, free volume, and segmental displacement under controlled thermal and mechanical conditions [34,35,36]. Yang et al. combined experimental characterization with molecular dynamics simulation and showed that non-bonded interaction evolution and internal free-volume redistribution are closely associated with tensile deformation and shape-memory recovery [37]. Wick et al. quantified energy-storage contributions in a thermoset shape-memory polymer with high recovery stress and suggested that conformational energy storage and release are important for thermomechanical recovery [38]. Hossain et al. showed that interchain non-bonded interactions strongly affect elastic response and yielding behavior [39], whereas Wu et al. reported a close correlation between free-volume evolution and deformation behavior in polymer systems [40]. Other molecular simulation studies have also indicated that free-volume growth, void nucleation, and cavity coalescence are important microscopic features during polymer deformation and recovery [41,42,43]. For PETG/POE thermo-responsive blends, systematic all-atom molecular dynamics studies remain limited. In particular, few studies simultaneously connect composition-dependent tensile deformation, cavity evolution, component-specific energetic interactions, and phase-resolved mobility across the fixed and recovery states. These considerations support the use of non-bonded interaction energy, free-volume and cavity descriptors, chain conformation, and mean square displacement (MSD) as useful molecular indicators for interpreting local thermomechanical behavior in amorphous polymer regions.

In this work, all-atom molecular dynamics simulations were performed to investigate the composition-dependent deformation and shape-memory behavior of PETG/POE blends. The simulated density and *T*_g_ were compared with available experimental data to assess the constructed models. Uniaxial tensile simulations were used to compare mechanical trends and deformation-induced free-volume evolution. Cavity number and maximum cavity volume were further quantified to describe cavity-like structural development during tensile deformation. Non-bonded interaction energies were decomposed into PETG-PETG, PETG-POE, and POE-POE contributions to evaluate component-specific energetic interactions. A programming-fixing-recovery cycle was simulated to examine shape fixity, shape recovery, chain conformational evolution, and energy variation. Finally, total and phase-resolved MSD analyses were performed at 200 and 450 K to compare segmental mobility in the fixed and recovery states. By combining component-specific energetic, cavity-related, and mobility descriptors, the present simulations extend the available experimental observations and support a molecular-level interpretation of composition-dependent stiffness, fixation, and thermal recovery in PETG/POE blends.

## 2. Atomistic Models and Simulation Methodology

### 2.1. Model Construction and Equilibration

**Figure 1:**
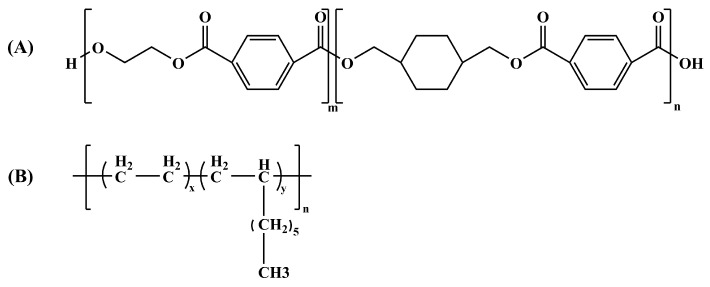
Molecular structures used for model construction: (**A**) PETG chain; (**B**) POE chain.

Atomistic models of PETG/POE blends were constructed using Materials Studio 2020 (MS) and subsequently equilibrated using LAMMPS 2026 [44]. PETG was represented as a random copolyester derived from terephthalic acid, ethylene glycol, and 1,4-cyclohexanedimethanol (CHDM). According to the experimental material specifications, poly(ethylene terephthalate) (PET) and poly(cyclohexanedimethylene terephthalate) (PCT) repeat units were incorporated at a molar ratio of 70:30 [26]. The cyclohexane ring in the CHDM unit was assigned the chair conformation. The degree of polymerization of each PETG chain was set to 30, corresponding to 966 atoms per chain. POE was modeled as a random ethylene/1-octene copolymer with a molar ratio of 85:15, consistent with the experimentally used Engage 8150 grade [26]. The degree of polymerization of each POE chain was set to 60, giving 830 atoms per chain. The molecular structures used for model construction are shown in Figure 1.

Periodic amorphous cells were generated using the Amorphous Cell module in MS, and periodic boundary conditions were imposed in all three directions. One pure PETG system and five PETG/POE blend systems with nominal PETG:POE mass ratios of 70:30, 60:40, 50:50, 40:60, and 30:70 were constructed [26]. Because a finite number of polymer chains was used in each simulation cell, the listed PETG/POE ratios should be regarded as nominal mass ratios approximating the target blend compositions. All initial amorphous cells were packed at an initial density of 0.60 g/cm^3^. The numbers of PETG and POE chains, total atom numbers, equilibrated box lengths, and equilibrated densities are summarized in Table 1.

The Polymer Consistent Force Field (PCFF) was used to describe bonded and non-bonded interactions [45]. The amorphous structures generated in MS were converted into LAMMPS-compatible data files, and the atom types, bonded topology, and molecular connectivity were checked before MD simulations. A cutoff distance of 10.0 Å was applied to short-range van der Waals and real-space Coulombic interactions, while long-range electrostatic interactions were treated using the particle–particle particle-mesh (PPPM) method with an accuracy of $1.0\times 10^{-4}$. Temperature and pressure were controlled using the Nosé-Hoover thermostat and barostat [46,47].

To remove unfavorable local contacts in the initially packed configurations, the systems were first energy-minimized using a steepest-descent step followed by conjugate-gradient minimization. A multistage equilibration protocol was then applied. Each system was relaxed in the NVT ensemble at 500 K for 45 ps, followed by NPT equilibration at 500 K and 1 atm for 200 ps to densify the initially loose amorphous packing. The systems were subsequently cooled from 500 to 298 K in the NPT ensemble over 200 ps and further equilibrated at 298 K and 1 atm for 100 ps. A time step of 0.5 fs was used during the first 5 ps of the initial high-temperature relaxation, and a time step of 1.0 fs was used for the remaining MD simulations.

**Figure 2:**
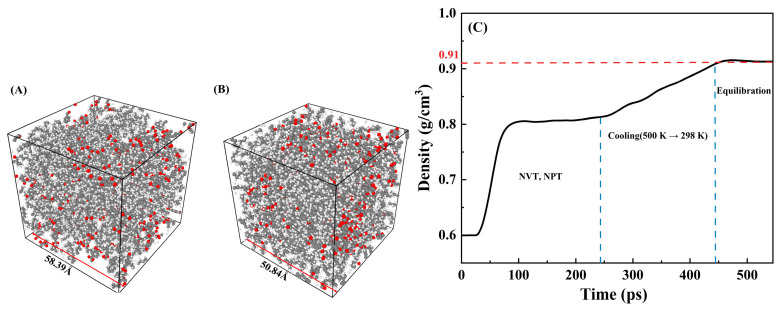
Equilibration of the representative PETG4/POE6 amorphous cell: (**A**,**B**) simulation cells before and after densification; (**C**) density evolution during the 545 ps equilibration process.

The equilibration process was assessed by monitoring the density evolution and inspecting the cell morphology. As illustrated by the PETG4/POE6 system in Figure 2, the simulation cell decreased in size during densification, and the density approached a stable value by the end of the 545 ps equilibration protocol. Visual inspection using OVITO 3.15.5 [48] showed no apparent large artificial voids at the simulation-cell scale. The equilibrated configurations were used as the starting structures for the subsequent glass-transition, tensile, and thermomechanical-cycle simulations. A more detailed discussion of the equilibrated densities and their comparison with reference data is provided in Section 3.1.

### 2.2. Simulation Details

#### 2.2.1. Glass Transition Temperature Determination

The glass transition temperature of each PETG/POE system was determined by non-equilibrium cooling simulations. For each composition, the equilibrated amorphous model obtained after the multistage relaxation procedure was used as the initial configuration. Before cooling, an additional energy minimization was performed using the conjugate-gradient algorithm to remove unfavorable local contacts and reduce residual internal stress.

The minimized structures were then cooled from 500 K to 200 K in the NPT ensemble at a pressure of 1 atm. The integration time step was set to 1 fs, and the total cooling time was 500 ps, corresponding to an average cooling rate of 0.6 K/ps. Temperature and pressure were controlled using the Nosé-Hoover thermostat and barostat, respectively. During the cooling process, the temperature, density, and simulation-cell volume were recorded every 10 ps.

The simulated *T*_g_ was extracted from the density-temperature relationship [49]. For each composition, two linear fits were applied to the approximately linear low-temperature glassy region and high-temperature rubbery region, while the transition region near *T*_g_ was excluded. The intersection of the two fitted lines was taken as the simulated *T*_g_ for the corresponding system.

#### 2.2.2. Uniaxial Tensile Simulation

Uniaxial tensile simulations of the PETG/POE blends were performed at 298 K with the loading direction set along the *x*-axis. Periodic boundary conditions were applied in all three directions, and a time step of 1 fs was used.

Before tensile loading, the equilibrated structures were subjected to energy minimization to remove unfavorable local contacts and residual stress. Each minimized system was then equilibrated in the NPT ensemble at 298 K and 1 atm for 100 ps to stabilize the room-temperature density, followed by an additional 100 ps equilibration in the NVT ensemble at 298 K to further relax the chain conformations.

Uniaxial deformation was then applied along the *x*-direction by continuously deforming the simulation box at a constant engineering strain rate of 1 × 10^−5^ fs^−1^, equivalent to 1 × 10^10^ s^−1^. During tensile deformation, the system temperature was maintained at 298 K using the Nosé-Hoover thermostat in the NVT ensemble, while the transverse box dimensions were kept fixed to maintain a well-defined and consistent uniaxial deformation protocol for all compositions. This setting also helped avoid numerical instability during large-strain deformation of the amorphous blend cells. The total loading time was 50 ps, corresponding to a final engineering strain of 50%. The possible influence of the high strain rate and fixed transverse dimensions on the tensile response and cavity evolution is further discussed in the limitations section.

The engineering strain was calculated using Equation (1):

(1) $$\begin{array}{c} \varepsilon =\frac{L_{x}-L_{0}}{L_{0}} \end{array}$$

where *L*_0_ and *L_x_* are the initial and instantaneous cell lengths in the x-direction, respectively. The tensile stress was obtained from the xx-component of the virial stress tensor. Young’s modulus was determined from the initial linear region of the stress–strain curve, and the yield stress was identified as the maximum stress before strain softening. The trajectories and thermodynamic data generated during 1loading were used for subsequent local-structure, free-volume, and energetic analyses.

#### 2.2.3. Shape-Memory Cycle Simulation

To quantify the thermally induced shape-memory response of the PETG/POE blends, the equilibrated systems were subjected to a nine-stage programming-fixing-recovery cycle. The high-temperature programming/recovery temperature was set to 450 K, and the temporary-shape fixing temperature was set to 200 K. A timestep of 1 fs was used throughout the simulations. The system was first heated from 298 to 450 K within 1 ns under an anisotropic NPT ensemble at 0.1 atm, followed by equilibration at 450 K for 1 ns under an NVT ensemble. The box length along the x-direction at the end of this stage was defined as *L*_0_.

Programming deformation was then imposed by stretching the system along the x-direction at 450 K under the NVT ensemble. A constant engineering strain rate of 1 × 10^−6^ fs^−1^ was applied for 0.5 ns, giving a total engineering strain of 50%, with the transverse box dimensions kept fixed. The deformed system was subsequently relaxed at 450 K for 1 ns under the NVT ensemble, and the corresponding *x*-direction box length was recorded as *L*_stretch_.

To fix the temporary shape, the stretched system was cooled from 450 to 200 K within 1 ns under the NVT ensemble and then relaxed at 200 K for another 1 ns. Unloading was performed by switching to an anisotropic NPT ensemble at 200 K and 0.1 atm for 1 ns, after which the *x*-direction box length was defined as *L*_freeze_. The system was then reheated from 200 to 450 K within 1 ns under the anisotropic NPT ensemble and further relaxed at 450 K for 3 ns to complete shape recovery. The final x-direction box length was recorded as *L*_recover_. The heating rate from 298 to 450 K was 0.152 K^ps^−1^, while the cooling and reheating rates between 450 and 200 K were 0.250 K^ps^−1^. The total duration of one shape-memory cycle was 10.5 ns.

Based on these characteristic lengths, the shape fixity ratio (*R*_f_) and shape recovery ratio (*R*_r_) were calculated according to Equations (2) and (3), respectively:

(2) $$R_{f}=\frac{L_{\mathrm{freeze}}}{L_{\mathrm{stretch}}}\times 100\%$$

(3) $$R_{r}=\frac{L_{\mathrm{freeze}}-L_{\mathrm{recover}}}{L_{\mathrm{freeze}}-L_{0}}\times 100\%$$

where *R*_f_ characterizes the ability of the system to maintain the temporary shape after unloading, and *R*_r_ represents the ability of the system to recover its original shape upon reheating. In the following sections, the shape-memory behavior of the different blend systems will be analyzed by combining dimensional evolution, energy variation, and chain conformational descriptors.

### 2.3. Analysis Methods and Molecular Descriptors

#### 2.3.1. Local Structure and Free-Volume Analysis

To characterize local packing changes associated with tensile deformation, radial distribution functions (RDFs) and free-volume descriptors were calculated from the MD trajectories. The RDF, g(r), was obtained using the coordination analysis module in OVITO for the equilibrated and deformed configurations [48]. This descriptor was used to evaluate short-range atomic packing and its variation during stretching.

The free volume was defined as the difference between the total system volume, *V*, and the occupied volume, *V*_a_, as defined in Equation (4):

(4) $$V_{f}=V-V_{a}$$

The corresponding free-volume fraction, *f*, was then calculated according to Equation (5):

(5) $$f=\frac{V_{f}}{V}$$

where *V*_f_ is the free volume. The free-volume fraction was used to quantify the unoccupied space in the amorphous blend and to monitor local packing loosening during tensile deformation.

Free-volume analysis was performed using the Construct Surface Mesh module in OVITO 3.15.5 together with a Python API script [50]. The probe radius was selected based on the nearest-neighbor distribution obtained from RDF analysis and trial calculations with different probe radii. A probe radius of 3.5 Å was used for all systems. This radius reduces spurious cavity detection in the initially equilibrated amorphous structures while retaining deformation-induced cavity-like free-volume regions. The calculated free-volume fraction and the spatial distribution of cavity-like regions were used in the subsequent tensile-deformation analysis. For the quantitative cavity analysis, cavity-like regions were identified from each tensile trajectory frame using the same probe radius and surface-construction settings. Connected cavity-like regions were counted as individual cavities, and the cavity volume of each connected region was recorded. The cavity number, maximum cavity volume, mean cavity volume, median cavity volume, and 90th-percentile cavity volume were then extracted as descriptors of cavity formation, growth, and coalescence during tensile deformation.

#### 2.3.2. Energy and Chain-Conformational Analysis

To analyze the energetic response during tensile deformation, the potential energy components of the PETG/POE blends were extracted from the MD trajectories. For the tensile simulations, the total potential energy is expressed in Equation (6):

(6) $$E_{\mathrm{total}}=E_{\mathrm{valence}}+E_{\mathrm{cross}}+E_{\mathrm{non}-\mathrm{bond}}$$

where *E*_valence_ is the valence energy, *E*_cross_ is the cross-term energy, and *E*_non-bond_ is the non-bonded interaction energy. The non-bonded contribution is further expressed in Equation (7):

(7) $$E_{\mathrm{non}-\mathrm{bond}}=E_{\mathrm{coul}}+E_{\mathrm{vdw}}$$

where *E*_coul_ and *E*_vdw_ are the Coulombic and van der Waals interaction energies, respectively. For comparison among different compositions, the energy values during tensile deformation were referenced to the zero-strain state. These quantities were used to track the evolution of intermolecular interactions under mechanical loading.

For the thermomechanical cycle, the *R_g_*, and the total energy were recorded at each stage to monitor chain extension, conformational retention, and recovery. The *R_g_* is an important descriptor of chain extension and was calculated according to Equation (8):

(8) $$R_{g}=\sqrt{\frac{1}{M}\overset{N}{\underset{i=1}{\sum }}m_{i}{\left(r_{i}-r_{cm}\right)}^{2}}$$

where *M* is the total mass of the polymer chain, *N* is the number of atoms in the polymer chain, *m_i_* is the mass of atom *i*, *r_cm_* is the center-of-mass position of the chain, and *r_i_* is the position vector of atom *i*. The combined analysis of *R_g_* and total energy was used to describe chain conformational evolution during the thermomechanical cycle.

#### 2.3.3. Segmental Mobility and Interaction-Energy Analysis

To characterize the low-temperature fixed state and the high-temperature activated state, additional equilibrium simulations were performed for each composition at 200 and 450 K. At each temperature, the equilibrated structure was first relaxed for 100 ps in the NPT ensemble at 1 atm, followed by a 1 ns sampling run at the same temperature. The saved atomic coordinates from this sampling period were used for both MSD and component-specific interaction-energy analyses.

The MSD was calculated from the time evolution of atomic positions after removing the center-of-mass motion. In addition to the total MSD of each system, PETG- and POE-resolved MSD values were calculated separately in the blend systems to distinguish the mobility contributions of the two components. For each temperature, the first frame of the 1 ns sampling trajectory was used as the reference configuration.

Using the same 200 and 450 K sampling trajectories, the non-bonded interaction energies were further decomposed into PETG-PETG, PETG-POE, and POE-POE contributions. PETG and POE atom groups were defined according to the molecular composition of each blend system. The group-based non-bonded interaction energies, including van der Waals and electrostatic contributions, were calculated from the saved trajectory frames. The reported values correspond to the mean values over 101 evenly spaced frames, and the standard deviations over these frames were used to describe temporal fluctuations.

The MSD was calculated using Equation (9):

(9) $$\mathrm{MSD}(t)=\frac{1}{N}\sum_{i=1}^{N}{\left|r_{i}(t)-r_{i}(0)\right|}^{2}$$

where *N* is the total number of atoms included in the MSD calculation, **r***_i_*(*t*) is the position vector of atom *i* at time *t*, and **r***_i_*(0) is the initial position vector of the same atom. The MSD and interaction-energy descriptors at 200 K and 450 K were used to compare low-temperature constraints and high-temperature activation across the PETG/POE compositions.

## 3. Results and Discussion

### 3.1. Model Validation and Composition-Dependent Thermal Behavior

The equilibrated densities of the PETG/POE models are summarized in Table 1. Neat PETG shows the highest density of 1.17 g/cm^3^, whereas the density decreases gradually to 1.03, 1.00, 0.95, 0.91, and 0.88 g/cm^3^ for PETG7/POE3, PETG6/POE4, PETG5/POE5, PETG4/POE6, and PETG3/POE7, respectively. This decrease follows the increase in POE fraction and is consistent with the lower density of POE compared with PETG. Therefore, the density variation reflects the expected change in overall molecular packing caused by the composition difference.

The equilibration behavior of the representative PETG4/POE6 system is shown in Figure 2. After the multistage relaxation process, the simulation cell changes from the initially loose packing state in Figure 2A to a more compact amorphous structure in Figure 2B. The corresponding density evolution in Figure 2C shows that the density increases during densification and then approaches a stable plateau at the end of the 545 ps equilibration process. Similar density stabilization was observed for the other blend compositions. In addition, no obvious large artificial voids were observed in the equilibrated cells by OVITO visualization. These results indicate that the initial packing heterogeneity was effectively reduced and that the equilibrated structures were suitable for subsequent comparative simulations.

**Figure 3:**
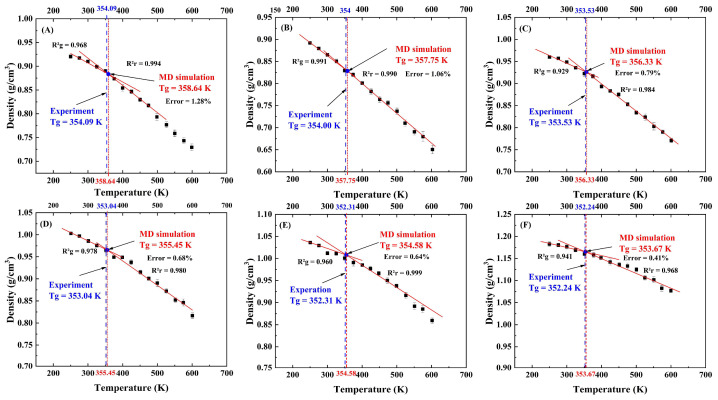
Comparison between simulated and experimental *T*_g_ values of the investigated systems: (**A**) PETG3/POE7; (**B**) PETG4/POE6; (**C**) PETG5/POE5; (**D**) PETG6/POE4; (**E**) PETG7/POE3; and (**F**) PETG.

The density-temperature relationships obtained during the cooling simulations are shown in Figure 3. The simulated *T*_g_ values were determined from the intersection of two linear fits to the approximately linear glassy and rubbery regions of the density-temperature curves, while the transition region near *T*_g_ was excluded. Because the simulated glass transition occurs over a finite temperature interval rather than at a sharp point, the fitting windows were selected according to the linear regions of each composition. The fitting quality was evaluated using the *R*^2^_g_ and *R*^2^_r_ values labeled in Figure 3. The obtained *T*_g_ values are close to the experimental differential scanning calorimetry (DSC) values reported by Cao et al. [26]. For example, the simulated *T*_g_ of PETG4/POE6 is 357.75 K, whereas the corresponding experimental value is 354.00 K. The relative errors between simulated and experimental *T*_g_ values are below 1.3% for all compositions. The simulated *T*_g_ values are slightly higher than the experimental values, which can be attributed to the much higher cooling rate used in MD simulations [51]. Under rapid cooling, segmental relaxation and conformational rearrangement are restricted within the simulation time scale, leading to a higher apparent glass transition temperature. Therefore, the simulated *T*_g_ values are expected to be cooling-rate dependent and are used here mainly to compare the composition-dependent trend rather than to precisely reproduce the experimental transition temperatures.

More importantly, the composition-dependent trend of *T*_g_ is reproduced. As the POE content increases, the overall *T*_g_ of the PETG/POE models increase, which is consistent with the experimental trend [26]. This behavior suggests that the incorporation of POE changes the local packing environment of the amorphous blends and affects segmental relaxation near the glass-transition region. The stable equilibration behavior, reasonable density variation, compact amorphous morphology, and consistent *T*_g_ trend support the use of the constructed PETG/POE models for comparative analysis. These results provide a reasonable structural and thermal basis for the following analysis of tensile deformation and thermomechanical recovery.

### 3.2. Composition-Dependent Tensile Response and Deformation Accommodation

#### 3.2.1. Tensile Stress Strain Response and Mechanical Parameters

**Figure 4:**
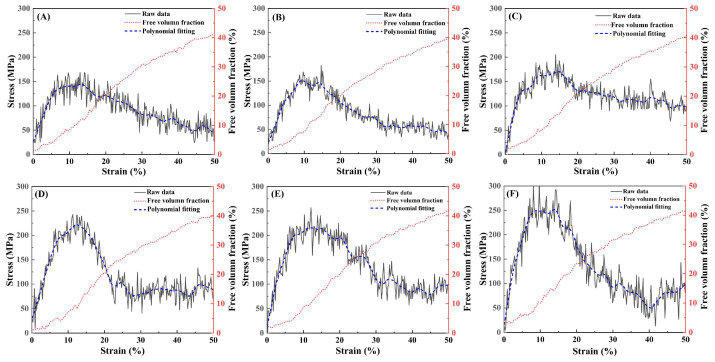
Stress–strain responses and free-volume fraction evolutions of the simulated systems under uniaxial tension: (**A**) PETG3/POE7; (**B**) PETG4/POE6; (**C**) PETG5/POE5; (**D**) PETG6/POE4; (**E**) PETG7/POE3; and (**F**) PETG.

The tensile stress–strain curves of neat PETG and PETG/POE blends are shown in Figure 4. All systems show a typical response of glassy amorphous polymers, consisting of an initial elastic region, yielding, and post-yield strain softening. At low strain, the stress increases almost linearly with strain, indicating that the deformation is mainly elastic. With further loading, the stress reaches a maximum and then decreases, corresponding to the onset of yielding and subsequent structural relaxation.

The mechanical parameters obtained from the stress–strain curves are summarized in Table 2. Neat PETG exhibits the highest Young’s modulus and yield stress, with values of 1.81 and 0.251 GPa, respectively. After POE incorporation, both Young’s modulus and yield stress show an overall decreasing trend with increasing POE content, despite minor variations between adjacent compositions. The Young’s modulus decreases from 1.78 GPa for PETG7/POE3 to 1.10 GPa for PETG3/POE7, while the yield stress decreases from 0.215 to 0.144 GPa. These results indicate that increasing the POE fraction lowers the stiffness and yielding resistance of the blends. Although the simplified high-strain-rate protocol may affect the absolute stress level, all compositions were simulated under the same loading conditions. Therefore, the stress–strain curves are interpreted mainly for comparing relative composition-dependent mechanical trends rather than for quantitatively reproducing experimental tensile stresses.

The decrease in stiffness and yield stress is consistent with the different chain characteristics of PETG and POE. PETG contains relatively rigid aromatic ester segments and contributes mainly to stiffness and load resistance, whereas POE has a flexible saturated backbone and allows easier chain rearrangement under tensile loading. Therefore, POE-rich systems deform at lower stress levels, while PETG-rich systems retain higher resistance to tensile deformation.

The simulated composition-dependent trend agrees with the experimental observation reported by Cao et al. [26], where increasing POE content leads to reduced tensile strength and increased elongation at break. The absolute Young’s modulus and yield stress obtained from MD simulations are higher than the experimental values, mainly because of the much higher strain rate and nanosecond time scale of MD simulations [39]. Therefore, the tensile results are used to compare relative composition-dependent mechanical responses rather than to quantitatively reproduce experimental stress values. This mechanical trend provides the basis for the following analysis of local packing loosening, free-volume evolution, and intermolecular interaction reorganization during tensile deformation.

#### 3.2.2. Local Packing and Free-Volume Evolution During Tensile Deformation

**Figure 5:**
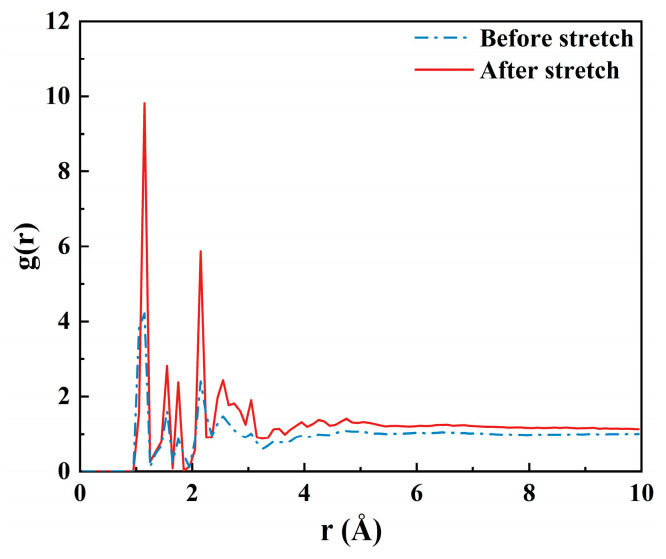
Radial distribution functions of the PETG4/POE6 system before and after uniaxial stretching.

The local packing response during tensile deformation was examined through RDF analysis. As shown in Figure 5, the RDF of the representative PETG4/POE6 system changes after stretching, particularly in the short-range region. The variation in peak intensity indicates that tensile loading induces rearrangement of local chain packing and perturbs the initial short-range order. This structural change is consistent with the transition from elastic loading to post-yield deformation observed in the stress–strain curves.

The free-volume fraction evolution in Figure 4 further shows that the amorphous structure gradually loosens during stretching. In the low-strain region, the free-volume fraction remains at a relatively low level, indicating that the chains are still densely packed and that local density fluctuations are limited. After yielding, the free-volume fraction increases more noticeably with strain, corresponding to the accumulation of locally loosened regions during plastic deformation. Therefore, the strain-softening stage is accompanied by the development of additional unoccupied space within the amorphous blend models.

The RDF and free-volume results describe different aspects of the same deformation process. RDF reflects short-range packing rearrangement, whereas free-volume fraction quantifies the increase in unoccupied space during stretching. Their combined evolution indicates that tensile deformation of PETG/POE blends involves local chain rearrangement, packing loosening, and enhanced density fluctuation. This packing response agrees with the composition-dependent tensile behavior of the blends.

#### 3.2.3. Cavity-like Free-Volume Regions and Energy Evolution

**Figure 6:**
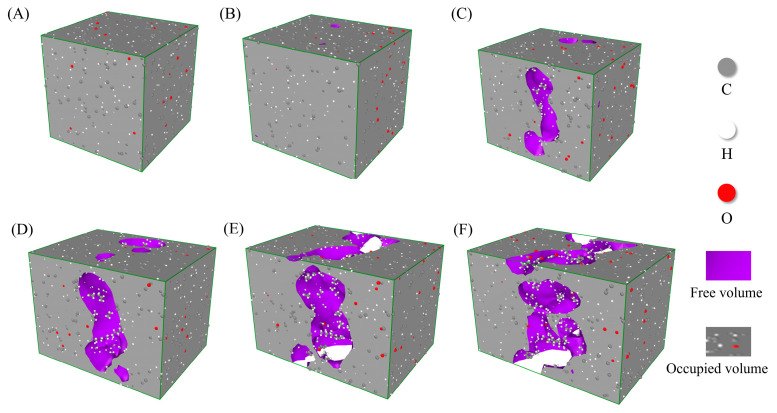
Evolution of cavity-like free-volume regions in the PETG4/POE6 blend during uniaxial tensile deformation: (**A**) ε = 0%; (**B**) ε = 10%; (**C**) ε = 20%; (**D**) ε = 30%; (**E**) ε = 40%; and (**F**) ε = 50%.

The cavity-like free-volume regions identified by the free-volume algorithm are shown in purple in Figure 6. At *ε* = 0, as shown in Figure 6A, no obvious purple regions are observed, indicating that the equilibrated structure is compact at the selected probe size. This result also indicates that the adopted 3.5 Å probe radius can distinguish the initially dense amorphous structure from the loosened regions generated during deformation.

At 10% strain, as shown in Figure 6B, sparse nanoscale cavity-like regions begin to appear, indicating the onset of local density fluctuation under tensile loading. As the strain increases to 20% and 30%, as shown in Figure 6C,D, these regions become larger and more numerous. At 40% strain, as shown in Figure 6E, neighboring cavity-like regions start to connect. At 50% strain, as shown in Figure 6F, an interconnected cavity-like free-volume network is formed. This progressive evolution indicates that tensile deformation induces not only an increase in free-volume content but also a redistribution of free volume within the amorphous structure, leading to a more heterogeneous local density field at large strain.

**Figure 7:**
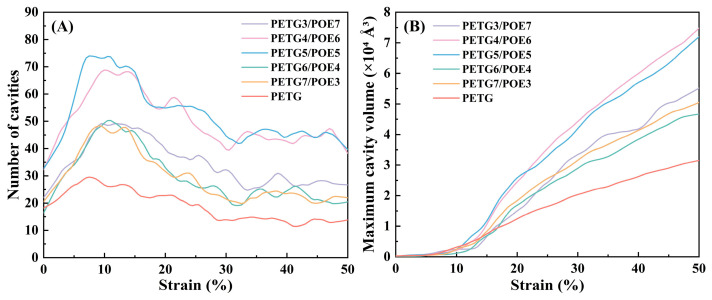
Quantitative evolution of cavity-like free-volume regions during tensile deformation: (**A**) number of cavities and (**B**) maximum cavity volume as functions of strain.

To further quantify the evolution of cavity-like free-volume regions, connected cavity-like regions were counted frame by frame from the tensile trajectories, and the number of cavities and maximum cavity volume were extracted, as shown in Figure 7 and summarized in Table 3. The number of cavities initially increases with strain, indicating the formation of multiple local low-density regions during the early stage of tensile deformation. For the blend systems, the maximum cavity number appears at approximately 9.0–14.5% strain, whereas neat PETG reaches its maximum cavity number at 7.5% strain. PETG4/POE6 and PETG5/POE5 show relatively higher maximum cavity numbers, suggesting more dispersed cavity formation in the intermediate-composition blends.

At larger strains, the cavity number decreases or fluctuates for several systems, whereas the maximum cavity volume continues to increase. This behavior suggests that some initially separated cavity-like regions gradually merge and grow into larger cavities during deformation. At 50% strain, the blend systems generally exhibit larger final maximum cavity volumes than neat PETG, indicating that POE incorporation facilitates cavity growth and local packing loosening under tensile deformation. Therefore, the cavity evolution is reflected not only by the increase in free-volume content, but also by the transition from dispersed small cavity-like regions to larger connected regions. These quantitative descriptors support the visual observations in Figure 6 and provide additional evidence for tensile-induced packing loosening, cavity formation, and cavity growth. The cavity and free-volume results are therefore interpreted together with the stress–strain response and energetic descriptors as comparative indicators of local deformation behavior under the same simulation protocol.

**Figure 8:**
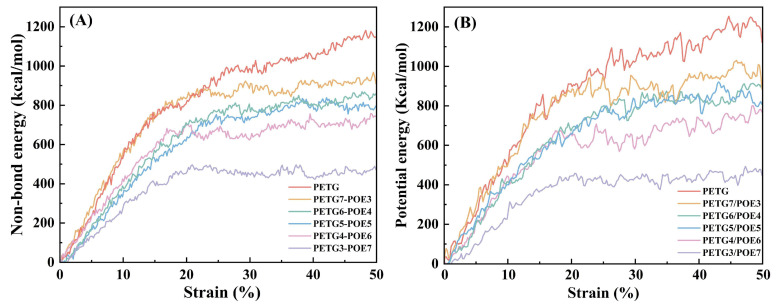
Evolution of (**A**) non-bonded energy and (**B**) total potential energy during tensile deformation for different simulated systems.

The energy evolution during stretching is shown in Figure 8. Both the non-bonded interaction energy and the total potential energy are plotted relative to the zero-strain state. With increasing strain, both energy terms increase continuously and follow similar trends, indicating that the energetic response during tensile deformation is closely related to the reorganization of intermolecular interactions. The rise in non-bonded energy mainly reflects the progressive separation and rearrangement of van der Waals and electrostatic interactions as the chains are stretched and locally displaced.

A comparison among different compositions shows that POE-rich systems exhibit a more gradual increase in non-bonded energy, whereas PETG-rich systems accumulate energy more rapidly. This behavior suggests that flexible POE chains facilitate local rearrangement and reduce the energetic cost associated with intermolecular separation. In contrast, PETG-rich systems retain stronger packing constraints and therefore show a larger energetic penalty under tensile deformation. The cavity-like free-volume evolution and energy variation link the composition-dependent tensile response of PETG/POE blends to local packing loosening and non-bonded interaction reorganization.

### 3.3. Shape-Memory Behavior and Molecular Mobility

#### 3.3.1. Thermomechanical Shape-Memory Cycle and Fixation-Recovery Behavior

**Figure 9:**
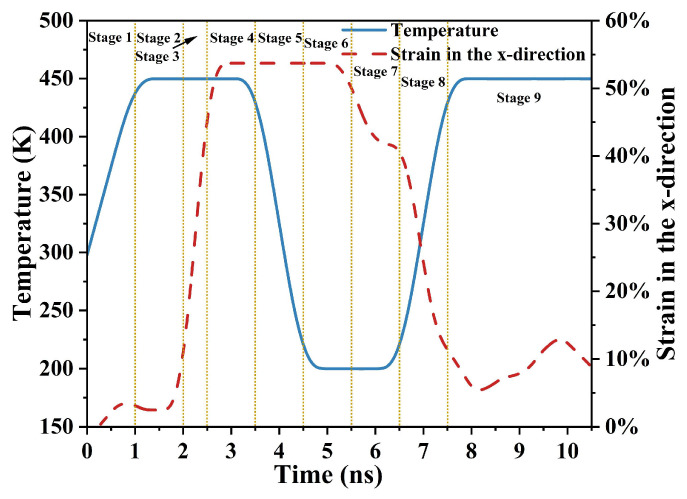
Thermomechanical shape-memory cycle of the PETG4/POE6 system.

The thermomechanical shape-memory cycle of the PETG/POE blends was simulated using PETG4/POE6 as a representative system, and the corresponding cycle curve is shown in Figure 9. The cycle was conducted with 450 K as the programming and recovery temperature and 200 K as the temporary-shape fixing temperature. The prescribed deformation was 50%, and one complete cycle lasted 10.5 ns. As shown in Figure 9, the coupled evolution of temperature and x-direction strain clearly separates the programming, fixing, and recovery stages.

During the programming stage, the system was heated from 298 K to 450 K, equilibrated at 450 K, and then stretched to 50% strain. The x-direction strain rapidly reached the programmed deformation level, indicating that the blend could be effectively deformed under high-temperature conditions. During the cooling and unloading stages, the deformed configuration was cooled to 200 K and then unloaded. Only limited strain recovery occurred after unloading, showing that most of the programmed deformation was retained at low temperature. During reheating and recovery, the system was reheated from 200 K to 450 K and further relaxed. The x-direction strain decreased progressively during this stage, indicating gradual release of the stored deformation and recovery toward the original state.

The simulated shape fixity and recovery ratios were compared with the experimental values reported by Cao et al. [26], as summarized in Table 4. The thermomechanical cycle in all-atom MD is necessarily accelerated because of the accessible simulation time scale. Thus, the heating, cooling, and recovery processes are much shorter than those in experimental shape-memory tests. The simulated *R*_f_ and *R*_r_ values are therefore not expected to exactly reproduce the experimental values, but they can be used as molecular-level indicators to compare the relative fixation and recovery behavior of different compositions under a consistent protocol.

In the simulations, neat PETG shows the highest *R*_f_ of 95.71% but the lowest $R_{r}$ of 75.17%. With increasing POE content, *R*_f_ gradually decreases to 90.48% for PETG3/POE7, whereas *R*_r_ increases to 84.43%. This trend indicates that increasing the POE fraction weakens the low-temperature locking ability of the blends but enhances their ability to release the programmed deformation during reheating.

Among the simulated compositions, PETG4/POE6 shows a relatively balanced fixation-recovery response, with an *R*_f_ of 92.45% and an *R*_r_ of 83.62%. Here, a balanced response refers to the simultaneous retention of high temporary-shape fixity and relatively high recovery capability within the same simulation protocol. According to this criterion, PETG4/POE6 does not exhibit the highest *R*_f_, which is observed for neat PETG, or the highest *R*_r_, which is observed for the POE-rich blend. However, it maintains both high fixity and relatively high recovery among the simulated compositions. Experimentally, PETG4/POE6 also shows balanced shape-memory performance, with an *R*_f_ of 90.07% and an *R*_r_ of 94.43% [26].

The lower simulated *R*_r_ of PETG4/POE6 compared with the experimental value is mainly attributed to the nanosecond-scale recovery stage in the MD simulation, which limits complete conformational relaxation within the simulated time window. Nevertheless, the consistent composition-dependent tendency, together with the chain snapshots, *R*_g_ evolution, and energy variation, supports the use of the cycle simulation to interpret the molecular origin of shape fixation and thermal recovery.

#### 3.3.2. Chain Conformational Evolution During Programming and Recovery

**Figure 10:**
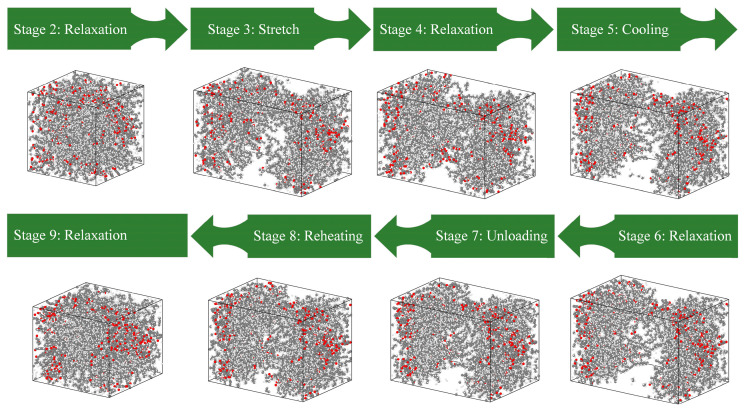
Representative snapshots of the PETG4/POE6 system at the end of Stages 2–9 during the shape-memory cycle simulation.

The representative chain conformations during the thermomechanical cycle are shown in Figure 10. After stretching at 450 K, the polymer chains become clearly oriented along the loading direction. During cooling to 200 K and subsequent unloading, most of the stretched conformation is retained, consistent with the high shape fixity observed in the cycle response. Upon reheating to 450 K, the chains gradually recoil from the extended state, indicating recovery of the programmed deformation. These snapshots directly illustrate the extension, retention, and recoil of the polymer chains during the cycle.

**Figure 11:**
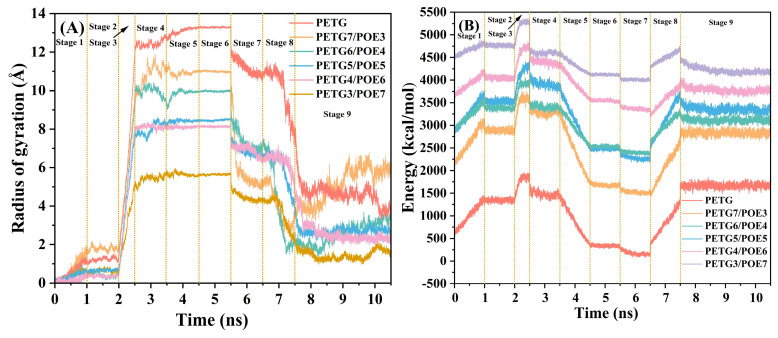
Evolution of (**A**) radius of gyration and (**B**) total energy during the shape-memory cycle simulation.

The conformational evolution is further quantified by the *R*_g_, as shown in Figure 11. During programming at 450 K, *R*_g_ increases as the chains are stretched and oriented under external loading. During cooling and low-temperature fixing, *R*_g_ remains at a relatively high level, showing that the extended conformations are largely preserved in the fixed state. During reheating and recovery, *R*_g_ decreases progressively, reflecting chain recoil and conformational relaxation toward the initial state.

The total energy in Figure 11 follows the same thermal-mechanical sequence. It increases during heating and high-temperature deformation, decreases during cooling to 200 K, and rises again upon reheating. This energy variation is consistent with the storage and release of deformation-related energy during the cycle. Together, the snapshots, *R*_g_, and total energy indicate that the shape-memory response is governed by reversible chain extension and recoil under temperature-dependent molecular mobility.

#### 3.3.3. Interaction-Energy Decomposition and Phase-Resolved Segmental Mobility

**Figure 12:**
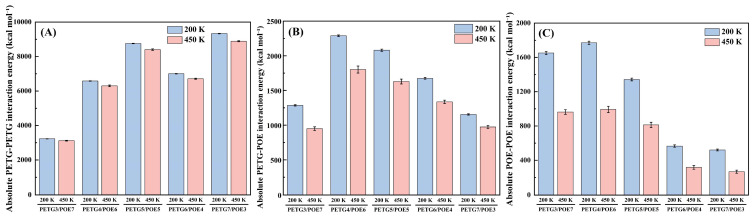
Absolute decomposed non-bonded interaction energies of PETG/POE blends at 200 and 450 K: (**A**) PETG-PETG, (**B**) PETG-POE, and (**C**) POE-POE. The original interaction energies are negative, indicating attractive non-bonded interactions; absolute values are plotted for clarity. Error bars represent standard deviations over 101 trajectory frames.

The component-specific energetic interactions were examined by decomposing the non-bonded interaction energies into PETG-PETG, PETG-POE, and POE-POE contributions, as shown in Figure 12A–C. All decomposed interaction energies are negative, indicating attractive non-bonded interactions. For clarity, their absolute values are plotted in Figure 12. Because these total interaction energies are influenced by both interaction strength and the number of atoms or contacts involved, they are used here as comparative energetic descriptors rather than direct measures of intrinsic pairwise interaction strength.

Among these terms, the PETG-PETG contribution gives the largest absolute values, reflecting the dominant cohesive interactions associated with the PETG-rich rigid phase. The PETG-POE contribution represents intercomponent interactions between the PETG and POE phases and is therefore particularly useful for evaluating composition-dependent intercomponent contact. As shown in Figure 12B, PETG4/POE6 and PETG5/POE5 exhibit relatively larger absolute PETG-POE interaction energies than the POE-rich and PETG-rich end compositions, suggesting more effective intercomponent contacts in the intermediate-composition blends. The POE-POE contribution becomes more evident in the POE-richer systems, consistent with the increasing role of the flexible POE phase.

For all three interaction-energy components, the absolute values at 450 K are lower than those at 200 K, indicating weakened non-bonded constraints at elevated temperatures.

**Figure 13:**
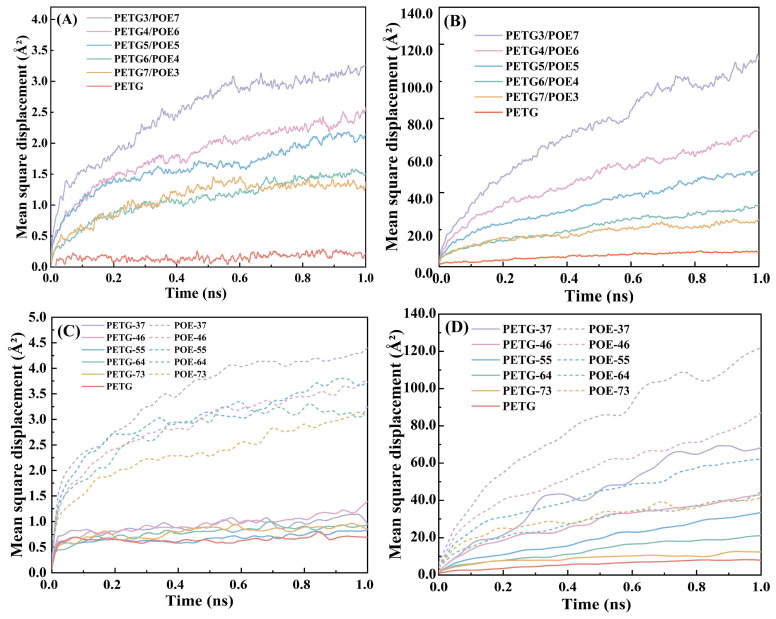
Mean square displacement curves of PETG and PETG/POE blends at (**A**) 200 K and (**B**) 450 K, and phase-resolved MSD curves of PETG and POE components in PETG/POE blends at (**C**) 200 K and (**D**) 450 K.

To further connect the energetic interactions with segmental mobility, the mobility of the PETG/POE blends was evaluated using total and phase-resolved MSD curves at 200 and 450 K, as shown in Figure 13. At 200 K, all systems show low MSD values and slow MSD growth, indicating strongly restricted chain displacement in the low-temperature fixed state. The phase-resolved MSD curves show that PETG atoms generally exhibit lower mobility than POE atoms in the blend systems. The differences among PETG components at 200 K are relatively small, suggesting that PETG chains remain strongly constrained in the glassy fixed state regardless of blend composition. Although POE shows higher MSD values than PETG, its absolute mobility remains limited at this temperature.

At 450 K, the MSD values increase markedly for all compositions, indicating thermally activated segmental motion. The mobility contrast between PETG and POE becomes more pronounced, with POE showing substantially higher MSD values than PETG in the blend systems. The POE-resolved MSD generally increases with increasing POE content, supporting the role of POE as the more mobile component during thermal activation. The PETG-resolved MSD also shows composition-dependent variation, with higher PETG mobility observed in POE-rich or intermediate blends than in neat PETG, suggesting that the flexible POE-containing environment can promote coupled segmental motion of PETG chains at elevated temperature.

Overall, the interaction-energy decomposition and phase-resolved MSD results provide complementary evidence for the fixation-recovery mechanism of PETG/POE blends. Strong PETG-related cohesive interactions and restricted PETG mobility contribute to low-temperature fixation, whereas enhanced POE mobility at elevated temperature facilitates conformational relaxation during recovery. Meanwhile, the relatively stronger PETG-POE interactions at intermediate compositions suggest more effective intercomponent contacts, which may help balance shape fixation and recovery under the present simulation protocol.

### 3.4. Scope and Limitations of the Present Simulations

The present all-atom MD simulations aim to provide molecular-level insights into the composition-dependent deformation and shape-memory behavior of PETG/POE blends. The constructed models were checked before comparative analysis. The density reached a stable plateau during equilibration. No obvious large artificial voids were observed in the equilibrated cells. The simulated density, *T*_g_, and composition-dependent mechanical trends were also consistent with available experimental data. These checks support the use of the models for comparative molecular-level analysis. However, because all-atom polymer blend simulations are computationally expensive, one representative amorphous cell was used for each nominal composition. Multiple independently generated replicas were not performed for all systems. Therefore, the numerical values should be interpreted mainly as comparative descriptors under the present simulation protocol, rather than statistically exhaustive material constants.

The finite atomistic cells represent local amorphous molecular environments. They do not represent mesoscale phase-separated morphologies. Therefore, the simulations are suitable for analyzing local packing, intermolecular interactions, cavity-like free-volume evolution, chain conformation, and segmental mobility. However, they cannot directly reproduce large-scale phase continuity, co-continuous morphology, or other mesoscale features observed experimentally.

The thermal protocols are also limited by the accessible MD time scale. The cooling rate used for *T*_g_ determination is much higher than that in experimental DSC measurements. The heating and cooling rates in the thermomechanical cycle are also much faster than experimental conditions. Therefore, the simulated *T*_g_ values are cooling-rate dependent. The simulated heating and cooling processes should be regarded as accelerated molecular-level representations, rather than direct reproductions of experimental thermal histories.

The mechanical protocols also involve simplifications. The tensile simulations were conducted at a high engineering strain rate of 1 × 10^−5^ fs^−1^, equivalent to 1 × 10^10^ s^−1^. The transverse box dimensions were kept fixed during tensile deformation. This setting was used to maintain a well-defined and consistent uniaxial deformation protocol for all compositions and to improve numerical stability during large-strain deformation. However, it may affect the absolute stress level, transverse relaxation, free-volume evolution, and cavity-growth kinetics compared with experimental tensile tests or simulations allowing lateral pressure relaxation.

The simulated programming-fixing-recovery cycle was performed over a nanosecond time scale. This time scale is much shorter than experimental recovery processes. Therefore, the simulated shape fixity and recovery ratios should not be regarded as direct quantitative predictions of experimental values. Instead, the cycle simulations are used to provide molecular-level insight into chain extension, conformational retention, and thermally activated recovery under a consistent simulation protocol. The quantitative results may also be affected by the employed force field and by the simplified representation of the polymer blend structure.

Accordingly, the simulated *T*_g_, stress–strain response, free-volume evolution, cavity-growth behavior, shape-memory parameters, interaction energies, and MSD results are used mainly for composition-dependent comparison and molecular-mechanism analysis. They should not be regarded as direct quantitative reproductions of experimental values. The conclusions therefore focus on how PETG/POE composition may affect local packing, intermolecular interactions, chain mobility, and conformational recovery under the present all-atom MD protocol. In addition, the present study discusses the fixation-recovery molecular mechanism of PETG/POE blends mainly on the basis of a representative thermomechanical cycle, and does not further evaluate performance degradation, fatigue accumulation, or failure limits under repeated cycling for different compositions. Therefore, the maximum number of shape-memory cycles and long-term cyclic durability as a function of composition still require further investigation through dedicated multi-cycle simulations combined with experimental validation. Future work using larger simulation cells, multiple independent replicas, slower thermal and mechanical protocols, longer recovery times, anisotropic lateral relaxation, improved force-field validation, and multiscale simulations would be valuable.

## 4. Conclusions

All-atom molecular dynamics simulations were performed to investigate the composition-dependent deformation and shape-memory behavior of PETG/POE blends. The equilibrated densities and simulated glass transition temperatures showed reasonable agreement with available experimental trends, supporting the use of the constructed models for comparative molecular-level analysis. Under the present simulation protocol, increasing POE content was associated with lower Young’s modulus and yield stress, consistent with the experimentally observed decrease in tensile strength and increase in elongation at break. Structural and energetic analyses further indicated that tensile deformation was accompanied by short-range packing rearrangement, free-volume growth, cavity-like free-volume development, density fluctuation, and non-bonded interaction reorganization. During the thermomechanical cycle, PETG-rich systems exhibited stronger temporary-shape fixation, whereas POE-rich systems showed enhanced recovery capability. Chain snapshots and *R*_g_ evolution supported chain extension during programming, conformational retention during fixing, and chain recoil during reheating. Phase-resolved MSD analysis further showed restricted PETG mobility at 200 K and enhanced POE mobility at 450 K, while the interaction-energy decomposition indicated strong PETG-related cohesive interactions together with relatively stronger PETG-POE intercomponent contacts at intermediate compositions. Overall, PETG contributes to stiffness and low-temperature fixation through stronger packing constraints and restricted chain mobility, while POE promotes deformation accommodation and thermally activated recovery by enhancing segmental motion. The structural, energetic, conformational, and dynamic descriptors link the experimentally observed fixation-recovery balance of PETG/POE blends to molecular packing, interaction reorganization, chain conformation, and segmental mobility. Among the investigated compositions, PETG4/POE6 shows a relatively balanced response, maintaining high shape fixity while retaining sufficient recovery capability. These findings provide molecular-level insight into the composition-dependent deformation and shape-memory behavior of PETG/POE blends.

## Figures and Tables

**Table 1:** Basic parameters of the simulated systems.

Model	Mass Ratio (PETG:POE)	Total Atoms	Equilibrated Box Length (Å)	Equilibrated Density (g/cm^3^)
PETG	100:0	4830	45.59	1.17
PETG7/POE3	70:30	8150	50.84	1.03
PETG6/POE4	60:40	8014	49.94	1.00
PETG5/POE5	50:50	12,300	43.29	0.95
PETG4/POE6	40:60	12,994	43.76	0.91
PETG3/POE7	30:70	9402	37.29	0.88

**Table 2:** Mechanical parameters of the simulated systems at 298 K.

Materials	PETG3/POE7	PETG4/POE6	PETG5/POE5	PETG6/POE4	PETG7/POE3	PETG
Young’s modulus (GPa)	1.10	1.16	1.59	1.80	1.78	1.81
Yield stress (GPa)	0.144	0.151	0.171	0.222	0.215	0.251

**Table 3:** Summary of cavity-like free-volume evolution during tensile deformation.

Composition	Initial Cavity Number	Max Cavity Number	Strain at Max Number (%)	Final Cavity Number	Final Void Fraction (%)	Final Maximum Cavity Volume (Å^3^)
PETG3/POE7	25	53	9.0	26	41.32	55,263.94
PETG4/POE6	36	77	14.5	43	39.61	74,759.91
PETG5/POE5	32	86	14.5	39	40.79	72,721.26
PETG6/POE4	14	57	9.5	20	40.09	46,928.65
PETG7/POE3	21	59	9.0	17	41.48	50,646.85
PETG	20	35	7.5	14	41.29	31,418.00

**Table 4:** Shape fixity ratio (*R*_f_) and shape recovery ratio (*R*_r_) of PETG/POE blends obtained from the shape-memory cycle simulations.

Simulation System	Simulated *R*_f_ (%)	Simulated *R_r_* (%)	Experimental *R*_f_ (%)	Experimental *R_r_* (%)
PETG3/POE7	90.48	84.43	80.67	97.27
PETG4/POE6	92.45	83.62	90.07	94.43
PETG5/POE5	92.61	78.88	92.59	92.07
PETG6/POE4	93.78	79.60	94.42	91.21
PETG7/POE3	94.91	78.30	100.00	85.67
PETG	95.71	75.17	100.00	82.43

## Data Availability

Data supporting the results of this study are available from the corresponding author upon reasonable request.
